# Assessment of risk of falls in elderly living at home**^1^**

**DOI:** 10.1590/1518-8345.0671.2754

**Published:** 2017-04-06

**Authors:** Adriana de Azevedo Smith, Antonia Oliveira Silva, Rosalina Aparecida Partezani Rodrigues, Maria Adelaide Silva Paredes Moreira, Jordana de Almeida Nogueira, Luiz Fernando Rangel Tura

**Affiliations:** 2 MSc.; 3 PhD, Associate Professor, Universidade Federal da Paraíba, João Pessoa, PB, Brazil.; 4 PhD, Full Professor, Escola de Enfermagem de Ribeirão Preto, Universidade de São Paulo, PAHO/WHO Collaborating Centre for Nursing Research Development, Ribeirão Preto, SP, Brazil.; 5 PhD, Researcher, Universidade Federal da Paraíba, João Pessoa, PB, Brazil.; 6 PhD, Adjunct Professor, Universidade Federal da Paraíba, João Pessoa, PB, Brazil.; 7 PhD, Professor, Universidade Federal do Rio de Janeiro, Rio de Janeiro, RJ, Brazil.

**Keywords:** Elderly, Accidental Falls, Risk

## Abstract

**Objective:**

to assess the risk of falls in elderly, by comparing the sociodemographic and cognitive factors, history of falls and self-reported comorbidities.

**Method:**

cross-sectional and quantitative study with 240 elderly. Data were collected based on the social profile, through the instrument of risk of falls and assessment of falls, by univariate analysis, bivariate and multiple logistic regression. The Statistical Package for the Social Sciences (SPSS) version 19 was used for statistical analysis.

**Results:**

there was a significant association of the risk of falls, as measured by the Fall Risk Score, with sex (<0.001), age (0.054), cognitive status (<0.001) and history of falls (<0.001). All variables were statistically significant and contributed to the occurrence of falls. In logistic regression, the variables that showed association with risk of falls were: fall, with whom they live, hypertension and visual impairment.

**Conclusion:**

female gender, older elderly (over 80 years old), with low cognitive status and occurrence of previous falls in the last six months are factors that increase the prevalence of falls. In logistic regression, the variables that were associated with risk of falls were: fall, with whom they live, visual impairment and rheumatologic diseases.

## Introduction

The increasing demographic aging observed in different countries is a complex phenomenon of global relevance that reflects in various sectors of society, including healthcare. 

Among the impacts and damages to the elderly's health, it was observed that the prevalence of chronic non-communicable diseases and external causes (such as falls and accidents), feature a significant magnitude and are major causes of morbidity and mortality^1^. In this context, it is important to note that the falls reach up to 32% of elderly people aged 65 to 74 years and 51% of elderly people over 85 years^2^. In a recent study, which covered a sample of 6,616 elderly residing in urban areas in 100 municipalities of 23 Brazilian states, it was observed that the prevalence of falls among them was 27.6%, in the 12 months prior to the interview^3^.

In the daily lives of the elderly, many factors can facilitate or promote the occurrence of falls. These factors are divided into two major groups: intrinsic, which are inherent to the person and related to the biological and psychosocial changes associated with aging; and extrinsic, which results from the interaction of the elderly with the environment, for example, quality of flooring and lighting in their residences, access to public transportation and recreational areas, among others. However, for being multifactorial events, these factors are related to the ability to maintain the skills needed to perform basic and instrumental activities of daily living, perceived as a requirement to live with independence and autonomy, so it is often difficult to report them separately^1^ ^,^ ^4^ ^-^ ^6^.

Thus, the high prevalence of falls can have serious consequences on the quality of life of the elderly, which can result in prolonged hospitalization, institutionalization, restriction of activities and mobility, changes in balance and postural control, social isolation, anxiety and depression^7^. In this way, it is important to know and identify the potentiating and protective factors, in order to adopt preventive measures for these events of falls. 

Therefore, changes resulting from the population aging have caused a new vision of care, which requires prioritization of the functional independence and autonomy. In this sense, the occurrence of falls interferes with biopsychosocial and economic aspects of the elderly and society, so it is important to prioritize the knowledge of risk factors and the impact of the occurrence of falls, with the aim of establishing preventive measures.

Given the above, the objective of this study was to evaluate the risk of falls among the elderly living at home, residing in the municipality of João Pessoa, Paraíba, Brazil.

## Methods

Cross-sectional study with a quantitative approach, carried out in the urban area of the municipality of João Pessoa, Paraíba, Brazil, through structured interviews with the elderly living at home, in the period from November 2010 to February 2011.

Participants were elderly over 60 years, of both sexes, residents in the selected census sectors, which were pre-selected among the 617 existing sectors used by the Brazilian Institute of Geography and Statistics (IBGE). For the draw of sectors, it was taken into account the number of elderly residing in the municipality of João Pessoa-PB, totaling 61,281, according to estimates by IBGE in 2007.

The self-weighted two-stage sample was calculated per conglomerate, through a process of probability sampling, and 20 census tracts were drawn with a probability proportional to the size of the sector, according to the 2000 census. Therefore, the Primary Sampling Unit (PSU) was the census tract, and the street was the unit drawn in the second stage. The sampling procedure occurred in two stages: setting up the same number of elderly people in the second stage, which ensures a self-weighted sampling, and therefore, each individual has a weight equal to one in the database. After the draw of the sectors, the street names were entered into the *Statistical Package for the Social Sciences (SPSS)* and a new draw was carried out by sector. Finally, the number of blocks was entered, and another draw was carried out. Data collection occurred in the opposite direction to the visited households, until reaching 12 elderly per sector, according to the study inclusion criteria. 

It was decided, therefore, on a sample of 240 individuals, which ensured a maximum error of 6.3% with 95% probability.

Later, data collection began in the homes of the elderly and the interviews lasted approximately 60 minutes, respecting the availability of time of each elderly.

Data collection was carried out in three stages. In the first stage, it was collected information on the identification, social profile and self-reported health problems, including the following variables: age (by age group); sex (male and female); ethnicity (white, brown and black); marital status (single, married, divorced, widowed); with whom the elderly live (with or without a partner, family or non-family); education (illiterate, low educational level - one to four years-, middle - five to eight years- or high - nine or more years); monthly income (no income, a minimum wage, two minimum wages, three to five minimum wages or more than six minimum wage) and morbidities self-reported by the elderly people with their respective medications prescribed by the doctor. For grading the cognitive status of the elderly, the second stage of data collection, the Mini Mental State Examination (MMSE)^8^ was used for the assessment of the cognitive status.

The third stage, concerning the assessment of the risk of falls, was measured by the Fall Risk Score instrument, which predicts falls in the elderly, regardless of their causes because it shows the association of the scores with the number of falls. The scale of risk of falls, previously validated for the Brazilian population^9^, uses five criteria to assess the risk of falls, which are: 1) the presence or absence of previous falls; 2) medications used by the elderly with their respective names; 3) presence or absence of sensory deficit; 4) mental status, through the Mini-Mental State Examination; 5) pace, assessing the way to walk. This scale ranges from zero to eleven points and scores ≥ three points suggest that the elderly has a high risk of falls^10^.

Data were analyzed using SPSS (Statistical Package for the Social Science) for Windows, version 19. In univariate analysis, quantitative variables were investigated using measures of central tendency (mean and median), dispersion (Standard Deviation) and proportions for categorical variables. In bivariate analysis for qualitative variables, measures of association were carried out in contingency tables (x², prevalence ratio and odds ratio or prevalence of odds ratio), considering 0.05 as level of significance. For the final analysis of the main outcome - the risk of falls - multiple logistic regression was used with the following predictive variables: gender, age group, occurrence of falls, with whom they live and some of the most prevalent comorbidities, considering 0.05 as level of significance. 

The Research Ethics Committee of the University Hospital Lauro Wanderley, of the Federal University of Paraíba, approved the project in accordance with the rules and guidelines for research with human beings, under opinion number 0598/10.

Participants were informed about the research development and those who agreed to participate have signed the Informed Consent Form, in two copies, one copy was given to the elderly. 

## Results 

The results show that most interviewees are female (69.6%). The prevalent age group was from 70 to 74 years, which corresponds to 24.2%, with the range varying from 60 to 96 years, mean of 71.6 years, median of 71.0 and the Standard Deviation of ± 7.5. 

White ethnicity predominated among the female elderly (44.3%) and brown ethnicity predominated among elderly men (46.6%). Regarding marital status, most were widows and married men.

It is observed that women have schooling from 5 to 8 years of study (30.5%), followed by the illiterates (22.9%). As for men, 28.8% reported schooling from 1 to 4 years of study and 24.7% reported middle level of education (5-8 years of study). 

Most elderly people have income equivalent to one minimum wage (45.8%), followed by 3 to 5 minimum wages (17.5%). The minimum wage for the period in which the study was conducted was R$ 510.00 in 2010 and R$ 545.00 in 2011.

Among the elderly, only 4.6% live alone in their homes. The others (95.4%) live accompanied, and of these, 33.8% live with their sons and grandsons (tri-generational arrangements), 16.3% live with their spouse and sons, and 13.8% live with their spouse.

Among the variables highlighted in the study, marital status and monthly income were statistically significant. 

To assess the risk of falls, such risk was associated with the following variables: *gender, age group, cognitive status and occurrence of falls*. The relative risk or risk ratio (RR) and prevalence odds ratio or odds ratio (OR) were statistically significant at a p-value <0.05. All variables were statistically significant, as shown in Table 1. 

**Table 1:** Distribution of the elderly by sex, age group, cognitive status and occurrence of falls, associated to the risk of falls. João Pessoa-PB, Brazil, 2011 (n=240).

Variable		High risk of falls		Low risk of falls		Total		Prevalence Ratio (95%CI)	Prevalence of Odds Ratio OR (95%CI)	p*
		N	%	N	%	N	%	PR	POR	
Sex										
	Female	96	57.5	71	42.5	167	100	1.749	2.761	<0.001
	Male	24	32.9	49	67.1	73	100	1.229-2.488	1.551-4.914	
Age Group										
	60-79 years	95	47.3	106	52.7	201	100	0.737	0.502	0.054
	80 years or older	25	64.1	14	35.9	39	100	0.559-0.972	0.247-1.021	
Cognitive Status										
	With impairment	39	79.6	10	20.4	49	100	1.877	5.296	<0.001
	Without impairment	81	42.4	100	57.6	191	100	1.510-2.333	2.498-11.231	
Presented falls										
	Yes	38	73.1	14	26.9	52	100	1.675	3.509	<0.001
	No	82	43.6	106	56.4	88	100	1.329-2.112	1.783-6.906	

*χ2 test; Level of significance: p<0.05.

In this research, it was found that the prevalence of falls in females, 1.749, was higher than in males. The prevalence of low cognitive status increases the risk of falls, 1.877, compared to the elderly people who did not suffered falls. The prevalence of falls among elderly people who have suffered previous falls in the last 6 months, 1.675, was higher compared to those who have not suffered falls.

Table 2 shows the association of the prevalent comorbidities with the risk of falls. Among them, stand out the systemic arterial hypertension (SAH), visual impairment, spinal problems, osteoporosis and rheumatic diseases as predictive factors for the occurrence of falls. Among the assessed comorbidities, obesity was the only one that was not statistically significant. 

**Table 2:** Distribution of elderly according to the self-reported comorbidities most prevalent, in relation to the risk of falls. João Pessoa-PB, Brazil, 2011 (n=240).

Comorbidities		High Risk for Falls		Low Risk for Falls		Prevalence Ratio PR (95% CI)	Prevalence of Odds Ratio OR (95%CI)	p*
		N	%	N	%	PR	POR	
Anxiety/Panic Disorder								
	Yes	32	68.1	15	31.9	1.493	2.545	
	No	88	45.6	105	54.4	1.164-1.916	1.295-5.002	0.006
Osteoarthritis/arthrosis								
	Yes	52	64.2	29	35.8	1.501	2.400	
	No	68	42.8	91	57.2	1.178-1.913	1.381-4.169	0.002
Hearing impairment								
	Yes	29	74.4	10	25.6	1.642	3.505	
	No	91	45.3	110	54.7	1.293-2.086	1.622-7.575	0.001
Diabetes Mellitus								
	Yes	35	62.5	21	37.5	1.353	1.941	0.033
	No	85	46.2	99	53.8	1.047-1.747	1.051-3.586	
Depression								
	Yes	29	82.9	6	17.1	1.867	6.055	<0.001
	No	91	44.4	114	55.6	1.506-2.314	2.410-12.212	
Arterial hypertension								
	Yes	98	67.6	47	32.4	6.919	2.918	<0.001
	No	22	23.3	73	76.8	3.835 -12.482	1.989-4.282	
Obesity								
	Yes	9	64.3	5	35.7	1.309	1.865	0.271
	No	111	49.1	115	50.9	0.867-1.977	0.606-5.738	
Osteoporosis								
	Yes	37	62.7	22	37.3	1.368	1.986	0.025
	No	83	45.9	98	54.1	1.062-1.760	1,086-3.630	
Spine Problems								
	Yes	60	60.0	40	40.0	1.400	2.000	0.009
	No	60	42.9	80	57.1	1.091-1.797	1.187-3.370	
Visual impairment								
	Yes	72	68.8	33	31.4	1.929	3.955	<0.001
	No	48	35.5	87	67.5	1.485-2.505	2.300-6.801	

*χ2 test; Level of significance: p<0,05.

It was also noted that elderly people affected by SAH (p <0.001) present a risk of falls approximately seven times higher than those who are not affected by this morbidity. Having visual deficit (p <0.001) and depression (p <0.001) increases in 1.929 and 1.867 times, respectively, the chances of falls. 

Other comorbidities were asked during this study; however, it is not emphasized those little reported. 

Table 3 shows the logistic regression results, which were achieved through saturated regression model, several predictors were analyzed such as *sex, age group, occurrence of falls, with whom they live and the four most prevalent comorbidities: SAH, visual impairment, spine problems and rheumatic diseases.* The variables associated with the risk of falls were occurrence of falls, with whom they live, visual impairment and rheumatologic diseases*.* As for the occurrence of falls, prevalence of high risk, 2.831, was higher than low risk. 

**Table 3:** Logistic regression analysis including the risk of falls and predictors. João Pessoa- PB, Brazil, 2011 (n=240).

Variable		High risk of falls		Low risk of falls		Prevalence of Odds Ratio	p*
		N	(%)	N	(%)	POR	
Sex							
	Female	96	57.5	71	42.5	1.248	0.609
	Male	24	32.9	49	67.1	0.534-2.916	
Age group							
	60-79 years	95	47.3	106	52.7	0.502	0.061
	80 years or older	25	64.1	14	35.9	0.244-1.033	
Presented falls							
	Yes	38	73.1	14	26.9	2.831	0.014
	No	82	43.6	106	56.4	1.234-6.496	
With whom they live							
	Alone	2	18.2	9	81.8	7.273	0.038
	Accompanied	71	31.0	158	69.0	1.113-47.547	
Morbidity							
	Arterial hypertension						
	Yes	98	67.6	47	32.4	1.748	0.135
	No	22	23.3	73	76.8	0.841-3.631	
Visual impairment							
	Yes	72	68.6	33	31.4	8.189	<0.001
	No	48	35.6	87	67.5	4.079-16.442	
Spine Problems							
	Yes	60	60.0	40	40.0	1.180	0.629
	No	60	42.9	80	57.1	0.603-2.308	
Rheumatic diseases							
	Yes	52	64.2	29	35.8	4.200	<0.001
	No	68	42.8	91	57.2	2.166-8.144	

*Multiple logistic regression; Level of significance: p <0.05.

The remaining predictors were statistically significant when evaluated separately, however, they were not statistically significant in logistic regression.

## Discussion

Falls are frequent events, however, for being multifactorial, it is difficult to establish a single risk factor for their occurrence. Thus, the risk of falls was associated with demographic and cognitive factors, occurrence of falls and self-reported comorbidities. 

In a study on the assessment of the prevalence and factors associated with falls in 2,096 individuals aged over 65 years in various states of Nigeria, it was observed sociodemographic characteristics, visual impairment, chronic physical conditions and insomnia. It was found that the major risk factors for falls were female gender, aged above 80 years, 7-12 years of study and low or medium socioeconomic status^11^.

Other studies have found greater propensity of falls among females compared to males and this difference is explained in terms of the physiological characteristics and bone and muscle structure, hormonal changes associated with menopause, as well as performing multiple tasks^9^ ^,^ ^12^ ^-^ ^13^. 

In this context, an observational and multicentric study conducted in the United States and Europe with 7,897 women diagnosed with post-menopausal osteoporosis, undergoing treatment at the time or prior to the survey, assessed which factors interfered in the quality of life of these women and found great impact of fear to fall on the quality of life^14^. 

In this study, it was observed that advanced age, higher consumption of medications and poor perception of the health status of the elderly, were factors that predisposed the falls, considering that be a male and literate are protective factors for these events. These results were consistent with a study conducted in the coverage area of basic health units, in 41 municipalities in seven Brazilian states, with a sample of 4,003 subjects over 65 years old. Such study showed a higher prevalence of falls associated with advanced age, sedentary lifestyle, self-perception of health status as poor and higher number of medications of continuous use^1^. 

On the other hand, literature shows that the risk of falls increases in subjects with cognitive impairment and score from 24 to 30 points in the Mini-Mental State Examination. These data are similar to those found in the present study, which occur due to visual-spatial disorientation, leading to a poor perception of environmental risks and an erroneous assessment of their own skills^13^. Therefore, a study carried out in the United States^15^ with 175 elderly over 65 years old, with high risk of falls and living in a community, assessed whether cognitive deficits increased the risk of falls. The authors concluded that there is an association between cognitive decline and increased risk of these events. In this context, it is worth noting that the cognitive assessment through the MMSE was based on the same score used by Bertolucci et al^8^, whose study carried out in 1994, has established the following cutoffs: 13 points for illiterate elderly people, 18 points for the elderly with 1 to 7 years of study and 26 points for the elderly with 8 or more years of study.

Among the risk factors related to the individual (Intrinsic) and associated to the events of falls discussed in the literature, is the decrease of sensory function, which is necessary for postural control, and whose components are: decreased visual capability and hearing impairment. Both are statistically related to the risk of falls, and in association with vestibular and proprioceptive disorders result in the reduction of the information on the axial balance and consequently, in increased reaction time to dangerous situations^16^. 

Other factors that may contribute to the falls are the changes in the Central Nervous System (CNS) and the musculoskeletal system disorders that accompany the aging process. These physiological events lead to a reduction in the density of the long bones and spine, with alterations in bone mineral balance and even more severe reductions, which may result in osteopenic and osteoporotic tissues^12^ also observed in the present study.

Studies reported in the literature and conducted with individuals in the community, have suggested that elderly patients who experienced previous falls are at increased risk of recurrence of these episodes, especially the elderly aged over 80 years, living alone, with complaints of dizziness, depressive symptoms and arthritis^17^ ^-^ ^18^. 

Multiple comorbidities may also favor the occurrence of falls as reported in the literature, since the prevalence of chronic diseases increases the possibilities of this event and drug interactions due to the use of multiple drugs^18^ ^-^ ^19^. Drug interactions (3 or more per day) or increased doses of psychoactive medications are associated with an increased number of hospitalizations due to falls. 

Other factors that may contribute to the risk of falls are depression and change in cognitive status because they reduce the willingness to carry out tasks, which causes muscle weakness and culminates with difficulty of walking^20^ ^-^ ^21^. Another author points out that patients with depression need to use medications such as the benzodiazepines, which may also contribute to the occurrence of falls in elderly^9^.

In a study on the factors associated with chronic diseases, with 385 elderly patients attended in the Family Health Strategy in Teófilo Otoni/Minas Gerais, found a significant prevalence of SAH (69.9%). In addition, 20% of the elderly have reported musculoskeletal problems^22^. Another study evaluating visual impairment, chronic physical conditions and insomnia in 2,096 individuals aged over 65 years in various states of Nigeria, found that the prevalence of falls increased in the following conditions: arthritis, pain in the thoracic and cervical spine and other pains, visual impairment and insomnia^11^. These results are relevant and corroborate the present study. 

Articular diseases are common in the elderly and may contribute to immobility, resulting in pain and postural imbalance, favoring the occurrence of falls^19^.

In this study, logistic regression through saturated regression model showed that the variables associated with the risk of falls were: fall, with whom they live, SAH and visual impairment. These findings were corroborated by a study conducted in China, which found a prevalence of 18% of falls and these were associated with the age range between 60 and 70 years old, female gender, decreased physical activity, visual impairment, living alone and with health problems, such as *diabetes mellitus* ^23^. 

Although this study has emphasized the sociodemographic characteristics and intrinsic factors of falls (inherent to the individual), the factors related to the environment should also be prioritized, since the occurrence of these events due to environmental inadequacies, can be minimized with the adoption of a small number of measures^24^. 

## Conclusion

The results of this study lead to the following conclusions: (1) The association of the risk of falls, measured by Fall Risk Score, with sex, age, cognitive status and occurrence of falls, shows that all the variables presented statistical significance; (2) Be female, older elderly (above 80 years), with low cognitive status and have experienced previous falls in the last six months, increase the prevalence of falls; (3) The self-reported morbidities that were statistically related with the occurrence of falls were: hypertension, visual impairment, spinal problems, osteoporosis and rheumatic diseases; (4) in logistic regression through saturated regression model, the variables associated with the risk of falls were: fall, with whom they live, visual impairment and rheumatologic diseases*.*

Falls are associated with a number of factors, so the multidisciplinary knowledge is essential to provide information on the prevention and identification of elderly at risk, justifying the need for this study. In this context, further studies of this nature on a larger scale are needed, since the cross-sectional design of this study positively influences the behavior of health professionals, however, this type of study design does not allow its temporal sequence, and the exposure to the factors evaluated cannot be monitored directly, which characterizes the possible limitations of this study. 
